# Supplementary material for: Power calculator for detecting allelic imbalance using hierarchical Bayesian model
Source: BMC Res Notes. 2021 Nov 27;14:436. doi: 10.1186/s13104-021-05851-x (PMC8626927; doi:10.1186/s13104-021-05851-x)
Supplement: Supplementary file 1 — Additional file 1. Additional Methods. This file contains the section additional methods, in which we summarize the definition of the Bayesian model used in this work. The model has been previously published and described, and we provide in additional methods a brief summary just to facilitate readers. [file 13104_2021_5851_MOESM1_ESM.docx]

Additional methods for:

# Power Calculator for Detecting Allelic Imbalance Using Hierarchical Bayesian Model

Katrina Sherbina^1^, Luis G. León-Novelo^2^, Sergey V. Nuzhdin^3^, Lauren M. McIntyre^4^, Fabio Marroni^5^

^1^Quantitative and Computational Biology Section, University of Southern California, Los Angeles, CA 90046, USA

^2^Department of Biostatistics and Data Science, The University of Texas Health Science Center at Houston-School of Public Health, Houston, TX 77030, USA

^3^Molecular and Computational Biology Section, University of Southern California, Los Angeles, CA 90046, USA

^4^Genetics Institute and Department of Molecular Genetics and Microbiology, University of Florida, Gainesville, Florida 32603, USA

^5^Dipartimento di Scienze Agroalimentari, Ambientali e Animali, Università di Udine, Udine, 33100, Italy

## **Additional Methods**

### **Model description**

Let *g1* and *g2* be the two alleles of a diploid individual, respectively. For each gene or gene region, condition *i* and biological replicate (biorep) *k,* $x_{i,k}$ and $y_{i,k}$ are the number of reads that align better (or unambiguously) to allele *g1* and *g2,* respectively, while $z_{i,k}$ is the number of reads that map equally well (or ambiguously) to both alleles (Table S1).

**Table S1.** The expected number of reads ($\mu$) aligning better to allele *g1* than *g2*, $x_{i,k}$; better to allele *g2* than *g1*, $y_{i,k}$; or ambiguously, that is equally well to both alleles, $z_{i,k}$.

| $\boldsymbol{x}_{\boldsymbol{i,k}}$ | $\boldsymbol{y}_{\boldsymbol{i,k}}$ | $\boldsymbol{z}_{\boldsymbol{i,k}}$ |
| --- | --- | --- |
| $\mathbf{(1}/{\boldsymbol{\alpha}_{\boldsymbol{i}}}\mathbf{)}\boldsymbol{\beta}_{\boldsymbol{i}\mathbf{,}\boldsymbol{k}}\boldsymbol{r}_{\boldsymbol{i}\mathbf{,}\boldsymbol{g}\mathbf{1}}$ | $\alpha_{i}\beta_{i,k}r_{i,g1}$ | ${[(1-r_{i,g1})}/{\alpha_{i}}+(1-r_{i,g2})\alpha_{i}$] $\beta_{i,k}$ |

One important parameter in determining AI is the ability to correctly assign reads to an allele given that the read originated from that allele. We express this as the quantity $r_{i,g1}$ ($r_{i,g2}$), which is the probability of a read aligning to allele *g1* (*g2*) given that it came from that allele. Low values of these probabilities correspond to a high degree of ambiguously mapped reads, which occurs when there is little sequence divergence between the two alleles.

AI in condition *i* is measured by the parameter $\theta_{i}$ representing the proportion of reads originating from the allele *g1*, which that can be written as follows:

$$\theta_{i}=\frac{\mathbb{E(}{x_{i,k}}/{r_{i,g1})}}{\mathbb{E(}{x_{i,k}}/{r_{i,g1}+{y_{i,k}}/{r_{i,g2}})}}=\frac{1/\alpha_{i}}{\alpha_{i}+1/\alpha_{i}}$$

Notably, when $\theta_{i}$ is close to 0 , we have one extreme case of AI with all the reads originating from *g2*. When $\theta_{i}=0.5$, we have perfect allelic balance with 50% of the reads from each allele. With $\theta_{i}=1$, we are in the opposite direction of extreme AI with all the reads originating from *g1*. $\theta_{i}$ is a function of $\alpha_{i}$, which is also a measure of AI representing the ratio of reads mapping to *g1* over the reads mapping to *g2*. Consequently, $\alpha_{i}$ may vary from zero, when all reads map to *g2*, to infinity, when all reads map to *g1*. In the case of allelic balance, $\alpha_{i}=1$.

Finally, the model allows incorporating biological variability across conditions and replicates via the variable$\beta_{i,k}$. The ideal case is $\beta_{i,k}=1$ for all bioreps, which indicates that each biorep has the same variance.

### **Simulations**

The following null hypotheses are defined:

- 1. Allelic balance in condition 1, *i.e.* null *H1*: $\theta_{1}=$ 0.5 or equivalently $\alpha_{1}=$ 1.
  2. Allelic balance in condition 2, *i.e.* null *H2*: $\theta_{2}=$ 0.5 or equivalently $\alpha_{2}=$ 1.
  3. Level of AI is the same in both conditions, *i.e.* null *H3*:$\theta_{1}=\theta_{2}$ or equivalently $\alpha_{1}=\alpha_{2}$.

To test these hypotheses, three scenarios are defined (Figure 1):

1. H1, H2 and H3 are satisfied
2. H1 is satisfied, H2 and H3 are violated
3. H1 and H2 are violated, H3 is satisfied

Read counts were simulated under various scenarios assuming a negative binomial model with a dispersion of 50 and the mean (μ) defined for $x_{i,k}$, $y_{i,k}$ and $z_{i,k}$ as shown in Table S1. The full list of the simulation parameters is shown in Supplementary Table 2.

The simulations were designed varying $\theta_{1}$ and $\theta_{2}$ from 0.25 to 0.75 with step 0.05. Previous work has shown that the results for $\theta_{i}>$ 0.5 $(\alpha_{i}<1$) are symmetric to those for $\theta_{i}<$ 0.5 ($\alpha_{i}>1$) [20] so we focus here on the former set of $\theta_{i}$values.

$r_{i,g1}$ and $r_{i,g2}$were simulated to vary between 0.2 and 0.8 with step 0.05.

The number of bioreps was set to 3 for most simulations. When investigating the effect of varying number of bioreps on type I and type II error, the number of replicates was varied between 3 and 12.

The total number of allele specific reads was varied from 12 to 480,000. Allele specific reads are reads that map unambiguously in the simulation. Informative reads were equally distributed across bioreps.

For simplicity, all simulations were run assuming $\beta_{i,k}=1$ for all conditions and replicates.

### **Computing type I and type II error**

Under the extended simulation scenario, type I error is defined as the proportion of simulations for which the Bayesian evidence against allelic balance is less than 0.05 when simulations were performed under the null hypothesis. Three different null hypotheses are possible, as shown in Fig. 1. Hypothesis H1 and H2 are null when $\theta_{1}=$ 0.5 and $\theta_{2}=$ 0.5, respectively. H3 is null when $\theta_{1}=\theta_{2}$. H3 can be null even when both H1 and H2 are not null if both conditions are simulated with the same level of AI.

The power to detect AI within a condition or a difference in AI between conditions is the proportion of simulations for which the Bayesian evidence against allelic balance or equal levels of AI is less than or equal to 0.05 when simulations were performed under the not null hypothesis. Within a condition, the H1 (or H2) hypothesis is not null when $\theta_{1}$ ≠ 0.5 (or $\theta_{2}$ ≠ 0.5). When comparing AI between conditions, the H3 hypothesis is not null when one condition is simulated with allelic balance ($\theta_{1}$ = 0.5) and the other with allelic imbalance ($\theta_{2}$ ≠ 0.5). magnitudes of deviation from the null, which are measured as $\Delta AI$. Given $\theta_{0}=0.5$,${\Delta AI}_{1}=\frac{\left| \theta_{1}-\theta_{0} \right|}{\theta_{0}}$ for H1 ${\Delta AI}_{2}=\frac{\left| \theta_{2}-\theta_{0} \right|}{\theta_{0}}$for H2, and ${\Delta AI}_{3}$ = $\frac{\left| \theta_{2}-\theta_{1} \right|}{\theta_{1}}$ for H3. Deviations of $\Delta AI$ from the null are moderate, generally between 0.1 and 0.3, with a maximum of 0.5. The reader can easily verify using the given equations that $\Delta AI$=0.5 can be obtained, when $\theta_{0}$ = 0.5 and $\theta_{1}$ = 0.25. If $\theta_{0}$ = 0.5 and $\theta_{1}$ = 0.4, then $\Delta AI$=0.2, and so on.
