# Supplementary material for: Power calculator for detecting allelic imbalance using hierarchical Bayesian model
Source: BMC Res Notes. 2021 Nov 27;14:436. doi: 10.1186/s13104-021-05851-x (PMC8626927; doi:10.1186/s13104-021-05851-x)
Supplement: Supplementary file 3 — Additional file 3. Variation of type I error as a function of number of simulations, number of allele specific reads per bioreps and extent of deviation from allelic balance. [file 13104_2021_5851_MOESM3_ESM.pdf]

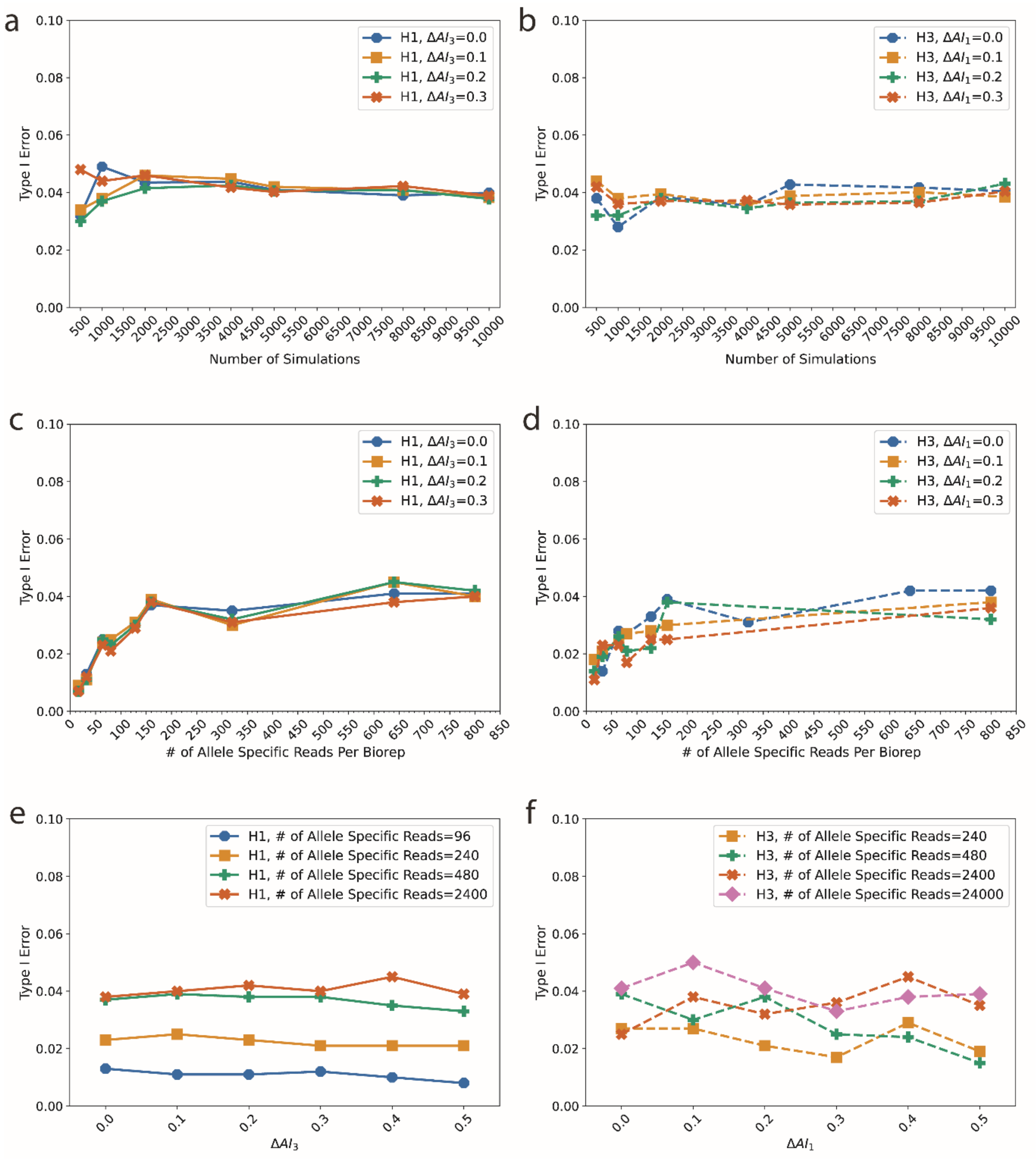

Type I error under different scenarios. (a-b) The x-axis is the number of simulations to obtain a read count dataset. The number (#) of allele specific reads was set to 2400, there were 3 bioreps, and the probability of an allele specific read was set to $r_{i,g1} = r_{i,g2} = 0.8$. Either ΔAI_1 or ΔAI_3 was varied from 0.5 to 0.65 by a step of 0.05 to test for H3 or H1, respectively. (c-d) The x-axis is the number (#) of allele specific reads per biological replicate (biorep). There were 1000 simulations and the probability of an allele specific read was set to $r_{i,g1} = r_{i,g2} = 0.8$. H1 and H3 were evaluated as for the simulations in a-b. (Bottom Left) H3 was evaluated using simulations of each of two conditions under the same $\theta \neq 0.5$. For H3, the effect size is the relative deviation from allelic balance in either of the two conditions $= \frac{|\theta - \theta_0|}{\theta_0}$, where $\theta_0 = 0.5$. In evaluating H1, the relative difference in the levels of allelic imbalance ΔAI was computed where the second condition was simulated under the not null hypothesis. (e-f) The x axis is the deviation from the null hypothesis of allelic balance in a condition ΔAI_1 or of equal levels of AI between conditions ΔAI_3. ΔAI_3 was varied to test for H1 while ΔAI_1 was varied to test for H3. There were 1000 simulations and the probability of an allele specific read was set to $r_{i,g1} = r_{i,g2} = 0.8$.
