# Supplementary material for: Power calculator for detecting allelic imbalance using hierarchical Bayesian model
Source: BMC Res Notes. 2021 Nov 27;14:436. doi: 10.1186/s13104-021-05851-x (PMC8626927; doi:10.1186/s13104-021-05851-x)
Supplement: Supplementary file 4 — Additional file 4. Variation of power as a function of number of bioreps. [file 13104_2021_5851_MOESM4_ESM.pdf]

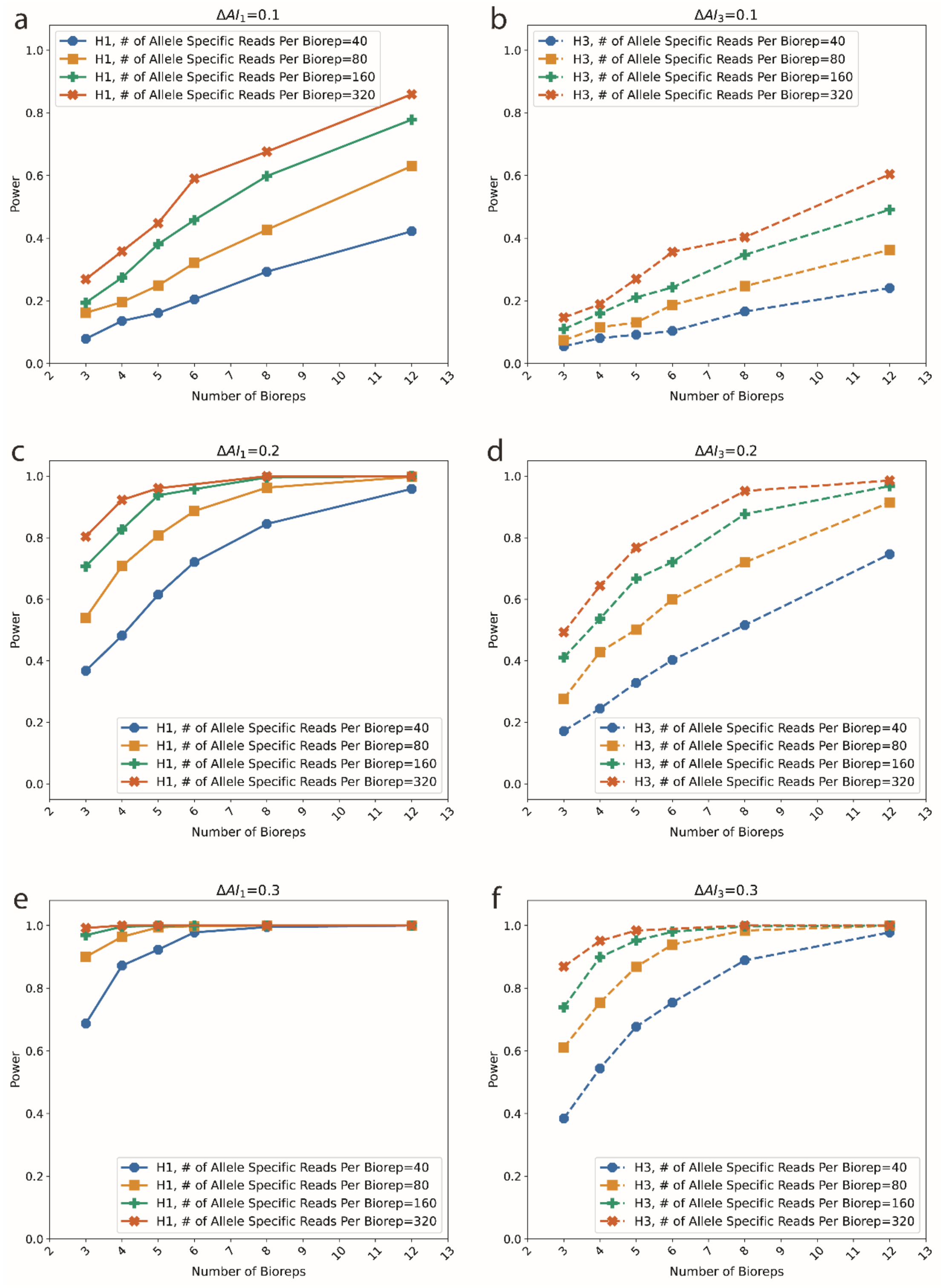

H1 and H3 refer to simulations under the not null hypothesis of allelic imbalance within a condition and unequal levels of AI between the two conditions, respectively. For evaluating H1, the x-axis is the effect size, which is the relative deviation from allelic balance in a condition $= \frac{|\theta-\theta_0|}{\theta_0}$, where $\theta_0 = 0.5$. For evaluating H3, the x-axis is the relative difference in levels of AI between two conditions $\Delta AI = \frac{|\theta_2-\theta_1|}{\theta_1}$ where the first condition simulated under the null hypothesis and the second under the not null hypothesis $\theta \neq 0.5$. The power (y-axis) is computed as the proportion of simulations for which the Bayesian evidence against allelic balance within a condition or against equal levels of AI between conditions is < 0.05. There were 1000 simulations and the probability of an allele specific read was set to $r_{i,g1} = r_{i,g2} = 0.8$. Simulations were performed for 3, 4, 5, 6, 8, and 12 biological replicates (bioreps, x-axis) for various number (#) of allele specific reads per biological replicate (bioreps). Increasing the number of biological replicates increases the power to detect AI or a difference in AI between conditions.
