# Supplementary material for: Power calculator for detecting allelic imbalance using hierarchical Bayesian model
Source: BMC Res Notes. 2021 Nov 27;14:436. doi: 10.1186/s13104-021-05851-x (PMC8626927; doi:10.1186/s13104-021-05851-x)
Supplement: Supplementary file 5 — Additional file 5. Variation of power as a function of number of simulations. [file 13104_2021_5851_MOESM5_ESM.pdf]

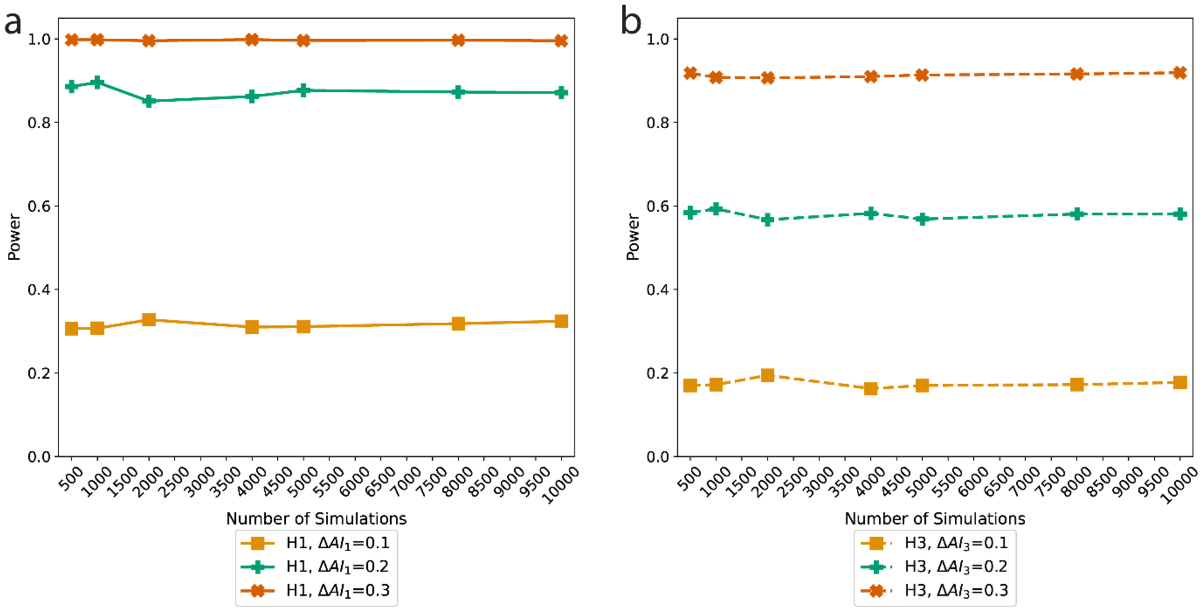

H1 and H3 refer to simulations under the not null hypothesis of allelic imbalance within a condition and unequal levels of AI between the two conditions, respectively. The x-axis is the number of simulations that were done to obtain a read count dataset. The power (y-axis) is computed as the proportion of simulations for which the Bayesian evidence against allelic balance within a condition or against equal levels of AI between conditions is < 0.05. In evaluating H1, the effect size is the relative deviation from allelic balance in a condition $= \frac{|\theta - \theta_0|}{\theta_0}$, where $\theta_0 = 0.5$. For H3, the relative difference in the levels of allelic imbalance $\Delta AI = \frac{|\theta_2 - \theta_1|}{\theta_1}$ was computed where the first condition and second condition were simulated under the null hypothesis of allelic balance and the not null hypothesis, respectively. The number (#) of allele specific reads was set to 2400 and the probability of an allele specific read was set to $r_{i,g1} = r_{i,g2} = 0.8$. At any given effect size or ΔAI, the power to detect AI in a condition or differing levels of AI between conditions is consistent across the number of simulations.
