# Supplementary material for: Power calculator for detecting allelic imbalance using hierarchical Bayesian model
Source: BMC Res Notes. 2021 Nov 27;14:436. doi: 10.1186/s13104-021-05851-x (PMC8626927; doi:10.1186/s13104-021-05851-x)
Supplement: Supplementary file 6 — Additional file 6. Variation of power as a function of number of allele specific reads per biorep. [file 13104_2021_5851_MOESM6_ESM.pdf]

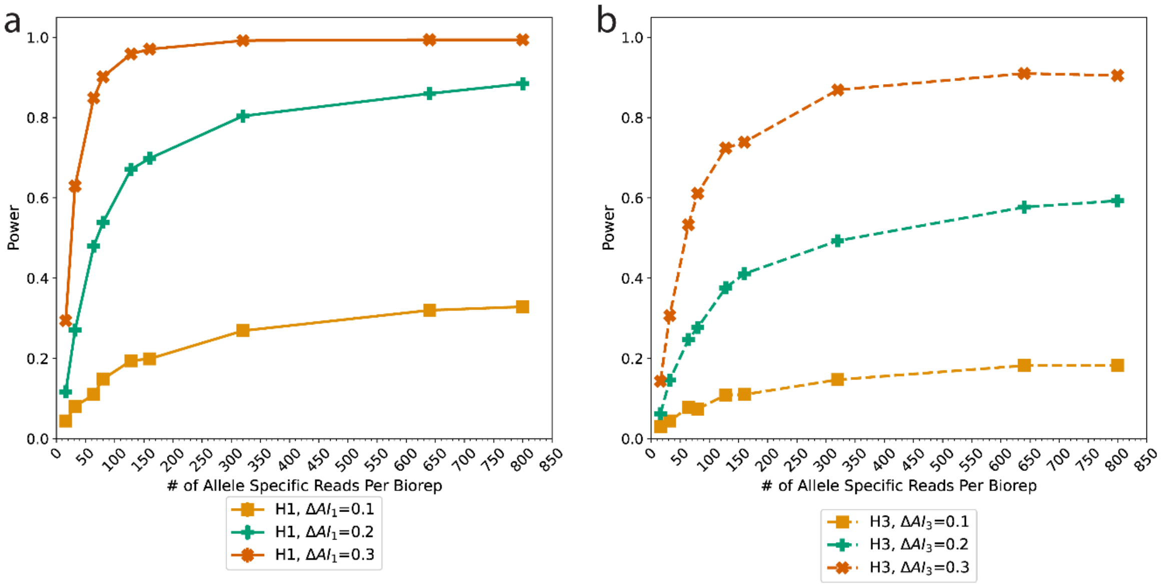

H1 and H3 refer to simulations under the not null hypothesis of allelic imbalance within a condition and unequal levels of AI between the two conditions, respectively. The x-axis is the number (#) of allele specific reads per biological replicate (biorep). The power (y-axis) is computed as the proportion of simulations for which the Bayesian evidence against allelic balance within a condition or against equal levels of AI between conditions is < 0.05. In evaluating H1, the effect size is the relative deviation from allelic balance in the condition $= \frac{|\theta - \theta_0|}{\theta_0}$, where $\theta_0 = 0.5$. For H3, the relative difference in the levels of allelic imbalance $= \frac{|\theta_2 - \theta_1|}{\theta_1}$ was computed where the first condition was simulated under the null hypothesis and second condition under the not null hypothesis. There were 1000 simulations, 3 biological replicates (bioreps) and the probability of an allele specific read was set to $r_{i,g1} = r_{i,g2} = 0.8$. The power to detect AI in a condition or differing levels of AI between conditions increases as the number of allele specific reads per biorep increases but does plateau for higher effect sizes and ΔAI.
